# Wedge resection vs. segmentectomy for lung cancer measuring ≤ 2 cm with consolidation tumor ratio > 0.25

**DOI:** 10.3389/fonc.2023.1253414

**Published:** 2023-08-28

**Authors:** Tetsuya Isaka, Takuya Nagashima, Hiroyuki Adachi, Hiroto Narimatsu, Kotaro Murakami, Shunsuke Shigefuku, Noritake Kikunishi, Naoko Shigeta, Kozue Watabe, Yujin Kudo, Yoshihiro Miyata, Morihito Okada, Norihiko Ikeda, Hiroyuki Ito

**Affiliations:** ^1^ Department of Thoracic Surgery, Kanagawa Cancer Center, Yokohama, Japan; ^2^ Department of Genetic Medicine, Kanagawa Cancer Center, Yokohama, Japan; ^3^ Cancer Prevention and Cancer Control Division, Kanagawa Cancer Center Research Institute, Yokohama, Japan; ^4^ Department of Surgery, Tokyo Medical University, Tokyo, Japan; ^5^ Department of Surgical Oncology, Hiroshima University, Hiroshima, Japan

**Keywords:** wedge resection, segmentectomy, propensity score matching, prognosis, overall survival, disease-free survival

## Abstract

**Objectives:**

We aimed to clarify the differences in prognosis between wedge resection and segmentectomy performed for cN0 non-small cell lung cancer (NSCLC) measuring ≤ 2 cm, with consolidation tumor ratio (CTR) > 0.25.

**Methods:**

This multicenter study included 570 patients with cN0 NSCLC (tumor size ≤ 2 cm, CTR > 0.25) who underwent wedge resection (n = 244) and segmentectomy (n = 326) between January 2010 and December 2018. After propensity score matching (PSM, 1:1 method), 182 patients were matched for clinical characteristics (age, sex, laterality, smoking index, tumor size, CTR, carcinoembryonic antigen value, positron-emission tomography-documented maximum standardized uptake value, clinical stage, and tumor disappearance rate) and intergroup comparison of disease-free survival (DFS) and overall survival (OS). Using Gray’s test, an intergroup comparison of the cumulative incidence of lung cancer-specific mortality was performed.

**Results:**

After PSM, similar DFS (5-year DFS, 79.9% vs. 87.1%, p = 0.103) and OS (5-year OS, 88.7% vs. 88.9%, p = 0.719) rates were observed in the wedge resection and segmentectomy groups. We observed no significant intergroup differences in lung cancer-specific mortality (5-year cumulative incidence: 4.6% vs. 3.5%; p = 0.235). Subgroup analysis revealed no specific subgroup demonstrating improved DFS or OS after undergoing wedge resection or segmentectomy.

**Conclusion:**

DFS, OS, and lung cancer-specific mortality were comparable between wedge resection and segmentectomy of cN0 NSCLC—tumor size ≤ 2 cm and CTR > 0.25. Large-scale prospective clinical trials are warranted to compare the prognoses of wedge resection and segmentectomy for these tumors.

## 1 Introduction

A randomized clinical trial comparing lobectomy and sublobar resection for early-stage lung cancer by the Lung Cancer Study Group in 1995 established lobectomy as the standard procedure for non-small cell lung cancer (NSCLC) of ≤ 3 cm in size and cN0 (1). However, with the widespread use of computed tomography (CT) in recent years, primary lung cancer has been detected at an earlier stage (2, 3), and the efficacy of sublobar resection for small peripheral lung cancers has been reported (4). Recently, large clinical trials of sublobar resection for early stage NSCLC have been conducted, and sublobar resection has been reported to be an acceptable alternative to lobectomy (5–8).

Sublobar resection can be classified as a segmentectomy or wedge resection. In JCOG0804/WJOG4507L, a single-arm study of sublobar resection for peripheral tumors 2 cm or smaller in diameter with consolidation tumor ratio (CTR) of 0.25 or less, the 5-year relapse-free survival (RFS) was 99.7% (5). In JCOG1211, a single-arm study of segmentectomy for tumor sizes of 2 cm to 3 cm with CTR of 0.50 or less, and tumor size 2 cm or less with CTR of 0.25 to 0.50, the 5-year RFS was 98% (6). In JCOG0802, a randomized clinical trial comparing overall survival (OS) between segmentectomy and lobectomy in patients with NSCLC of 2 cm with CTR > 0.5, segmentectomy was superior to lobectomy in terms of OS (7). Moreover, the recent CALGB140503 (Alliance) randomized clinical trial demonstrated the non-inferiority of sublobar resection for cN0 NSCLC ≤ 2 cm, excluding pure ground-glass nodules. This finding suggests that sublobar resection is a viable and acceptable treatment option for small-sized NSCLC (8). However, it remains unclear whether wedge resection is as effective as segmentectomy for NSCLCs ≤ 2 cm in size with CTR > 0.25.

Due to the longer operative time (9–11), increased blood loss (9, 11), and higher frequency of postoperative complications (9, 10, 12, 13), segmentectomy is a more invasive procedure than wedge resection for patients with lung cancer. Furthermore, segmentectomy is more technically challenging than wedge resection and requires a greater level of surgical expertise (9). However, wedge resection has been reported to have smaller tumor margins than segmentectomy (9, 10, 12, 14) and there is a difficulty in dissecting lymph nodes, especially in the hilar region (9, 11–13). There is concern that wedge resection may be less curative for tumors than segmentectomy (10, 15, 16).

Although there have been many reports comparing the outcomes of wedge resection and segmentectomy (9–11, 13–20), there have been only a few reports comparing the two surgical techniques for cN0 NSCLC ≤ 2 cm in size (4, 19, 21, 22). Therefore, there is still no consensus on the difference in therapeutic efficacy between wedge resection and segmentectomy for small tumors. It is also unclear whether wedge resection can be interpreted as an equivalent surgical technique to segmentectomy in the CALGB140503 study. This multicenter study compared the prognosis of wedge resection and segmentectomy in cN0 NSCLC patients ≤ 2 cm in size with CTR > 0.25 using propensity score matching (PSM) analysis.

## 2 Materials and methods

### 2.1 Ethics statement

The institutional review board of the participating institutions has approved this retrospective review of the multicenter database and waived the requirement for informed consent for each patient (Kanagawa Cancer Center, approval 24EKI54 (Approved on June 14, 2021); Tokyo Medical University Hospital, approval SH2969; Hiroshima University Hospital, approval E-1216).

### 2.2 Patients

**Figure 1:**
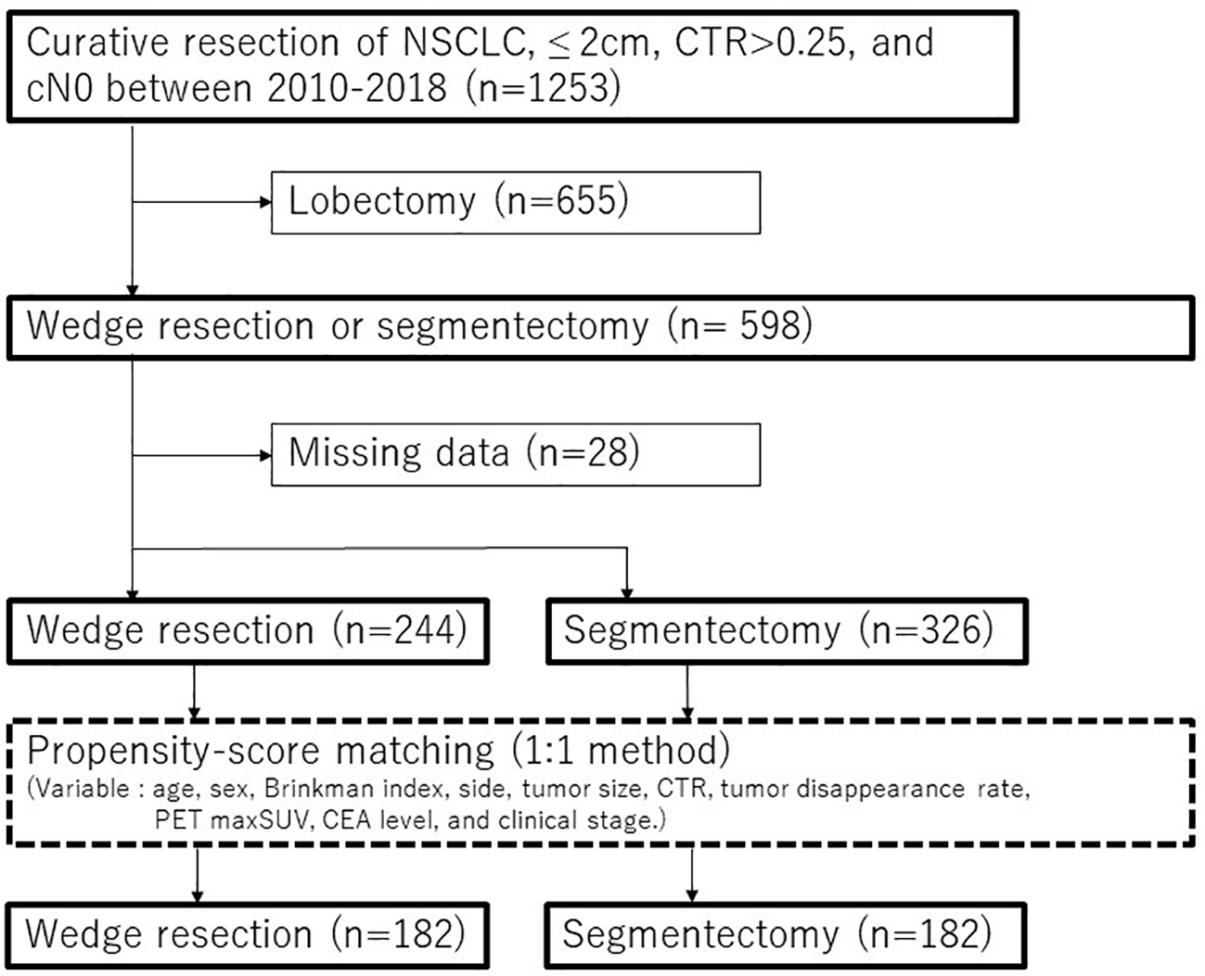
Consort diagram of this study. CEA, carcinoembryonic antigen; CTR, consolidation tumor ratio; NSCLC, non-small cell lung cancer; PET, positron emission tomography; maxSUV, maximum standardized uptake values.

Among the 1253 consecutive NSCLC patients who underwent complete resection for tumors ≤ 2 cm in size with CTR > 0.25 and cN0 from January 2010 to December 2018, 244 patients who received wedge resection and 326 patients who received segmentectomy were included in this study (**Figure 1**). Patients with NSCLC and pure ground-glass nodules were excluded. Segmentectomy was primarily carried out in patients with cN0 lung tumor ≤ 2 cm in size and had a CTR > 0.25. However, the decision to perform wedge resection was influenced by on surgeons’ preferences, considering factors such tumor location, radiological characteristics of the tumor, as well as patients’ age, comorbidities and lung function. TNM staging was performed according to the 8th edition of the TNM classification for lung and pleural tumors (23).

### 2.3 Word definitions

Patients who underwent a subsegmentectomy or two segmentectomies that were less than a lobectomy were included in the segmentectomy group. The study included patients who underwent segmentectomy, either with or without mediastinal lymph node dissection. The Brinkman index was defined as the number of cigarettes smoked per day multiplied by the number of years of smoking, as previously reported (24). Overall survival (OS) was defined as the period from the date of surgery to the date of death or censoring of patients without events during the last observation period. Disease-free survival (DFS) was defined as the period from the date of surgery to the date of recurrence or death from any cause; patients without recurrence were censored during the last observation period. Lung cancer-specific mortality was defined as death attributable to lung cancer in patients with postoperative recurrence. Intrathoracic recurrence included recurrence in the lungs, mediastinum, hilar and subclavian lymph nodes, and pleura, without extrathoracic recurrence. Extrathoracic recurrence included recurrence at sites other than intrathoracic sites, such as the bone, central nervous system, and abdominal organs.

### 2.4 Statistical analyses

The Mann-Whitney U test was performed to compare continuous variables between the wedge resection and segmentectomy groups, while Fisher’s exact test was used to compare categorical variables. All survival curves were analyzed using the Kaplan-Meier method and compared using the log-rank test. Univariable and multivariable analyses were performed using the Cox proportional hazard regression model to assess the impact of potential prognostic factor for DFS and OS with the following variables: age, sex, smoking history, CTR, positron emission tomography (PET) maximum standardized uptake values (maxSUVs), carcinoembryonic antigen (CEA) level (≤5ng/ml or >5ng/ml), histology (adenocarcinoma or non-adenocarcinoma), pleural invasion, and surgical procedure (wedge resection or segmentectomy).

To reduce selection bias between patients who underwent wedge resection and those who underwent segmentectomy, PSM analysis was conducted using a 1:1 matching method (caliper = 0.001). The patients were matched based on the following preoperative variables: age, sex, Brinkman index, side, CT tumor size, CTR, tumor disappearance rate, PET maxSUV, CEA level, and clinical stage (**Figure 1**). The cutoff values for age, CT tumor size, tumor disappearance rate, and PET maxSUVs were determined using receiver operating characteristic curve analysis. An inverse probability of treatment weighting (IPTW) analysis based on propensity scoring was also conducted as a supplemental. Multivariable analysis was performed on all variables with a p-value of < 0.05 in the univariable analysis. The cumulative incidence of lung cancer-specific mortality, intrathoracic recurrence, and extrathoracic recurrence was analyzed using Gray’s test. Statistical significance was set at p < 0.05. Statistical analyses were performed using EZR (Saitama Medical Center, Jichi Medical University, Saitama, Japan), which is a graphical user interface for R (The R Foundation for Statistical Computing, Vienna, Austria).

## 3 Results

**Figure 2:**
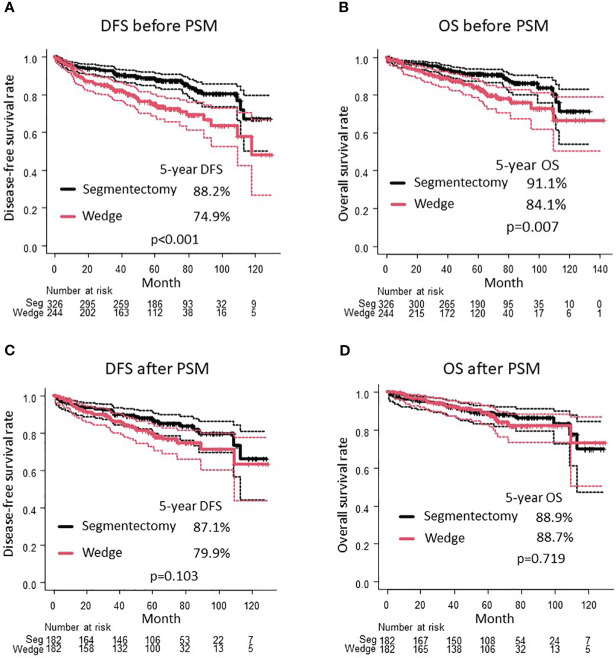
Comparison of DFS **(A)** and OS **(B)** between wedge resection group and segmentectomy group before PSM. Comparison of DFS **(C)** and OS **(D)** between wedge resection group and segmentectomy group after PSM. DFS, disease-free survival; OS, overall survival; PSM, propensity score matching.

The median observation period was 60.4 (42.1–77.9) months. Before PSM, patients in the wedge resection group were older (72 vs. 69 years, p < 0.001) and had more frequent pleural invasion (14.3% vs. 6.7%, p = 0.004), pathological T1c stage or higher (20.9% vs. 13.2%, p = 0.017), and recurrence (16.8% vs. 5.5%, p < 0.001) (**Table 1**). The wedge resection group had a worse DFS (5-year DFS 74.9% vs. 88.2%, p < 0.001; **Figure 2A**) and OS (5-year OS 84.1% vs. 91.1%, p = 0.007; **Figure 2B**) than the segmentectomy group.

**Table 1:** Comparison of clinicopathological characteristics between segmentectomy and wedge resection groups before propensity score matching.

Total n=570	Segmentectomy (n=326)	Wedge resection (n=244)	p value^a)^
Median age (IQR), y	69 (62-74)	72 (66-78)	<0.001^b)^
Male, No. (%)	167 (51.2)	139 (57.0)	0.176
Smoking history (+), No. (%)	175 (53.7)	143 (58.6)	0.268
Right side, No. (%)	147 (45.1)	128 (52.5)	0.090
CT Tumor size (IQR), cm	1.4 (1.2-1.7)	1.4 (1.2-1.7)	0.263^b)^
Tumor disappearance rate (IQR), %	30.5 (5-70)	33.5 (5-70)	0.622^b)^
CTR, No. (%) 26-50 51-99 100	88 (27.0) 90 (27.6) 148 (45.4)	58 (23.8) 69 (28.3) 117 (48.0)	0.679
PET maxSUV value (IQR)	1.22 (0.56-2.43)	1.12 (0-2.68)	0.462^b)^
CEA elevation, No. (%)	52 (16.0)	50 (20.5)	0.185
Clinical stage IA2≤. No. (%)	167 (51.2)	126 (51.6)	0.933
Ly (+), No. (%)	34 (10.4)	30 (12.3)	0.505
V (+), No. (%)	46 (14.1)	46 (18.9)	0.136
Pleural invasion, No. (%)	22 (6.7)	35 (14.3)	0.004
Pulmonary metastasis, No. (%)	4 (1.2)	4 (1.6)	0.730
Adenocarcinoma, No. (%)	282 (86.5)	196 (80.3)	0.051
Pathological T1c≤, No. (%)	43 (13.2)	51 (20.9)	0.017
Pathological stage IA3≤. No. (%)	50 (15.3)	51 (20.9)	0.096
Recurrence, No. (%)	18 (5.5)	41 (16.8)	<0.001
Intrathoracic only, No. (%)	17 (5.2)	32 (13.1)	0.001
Lung	10 (3.1)	15 (6.1)	0.097
Lymph node	6 (1.8)	8 (3.3)	0.288
Surgical margin	0	5 (2.0)	0.014
Pleura	4 (1.2)	8 (3.3)	0.138
Extrathoracic (and/or intrathoracic), No. (%)	1 (0.3)	9 (3.7)	0.003

a) Fisher’s exact test, b) Mann-Whitney U test.

CEA, carcinoembryonic antigen; CT, computed tomography; CTR, consolidation tumor ratio; IQR, interquartile range; Ly, lymphatic vessel invasion; maxSUV, maximum standardized uptake values; PET, positron emission tomography; V, blood vessel invasion.

In multivariable analysis using Cox proportional hazard regression model, age, sex, CTR, CEA value, histology, and pleural invasion were independent prognostic factors for DFS; however, wedge resection was not prognostic (hazard ratio [HR], 1.46; 95% confidence interval (CI), 0.99–2.16; p = 0.059) (**Table 2**). Moreover, age, sex, CEA value, histology, and pleural invasion were independent prognostic factors for OS; however, wedge resection was not prognostic (HR, 1.14; 95% CI, 0.71–1.81; p = 0.590) (**Table 2**).

**Table 2:** Univariable and multivariable analyses of disease-free survival and overall survival.

Variable	Disease-free survival						Overall survival					
	Univariable analysis			Multivariable analysis			Univariable analysis			Multivariable analysis		
	HR	95% CI	p value	HR	95% CI	p value	HR	95% CI	p value	HR	95% CI	p value
Age	1.07	1.04-1.09	<0.001	1.05	1.02-1.07	<0.001	1.10	1.07-1.14	<0.001	1.09	1.06-1.12	<0.001
Sex (male)	3.16	2.03-4.89	<0.001	2.18	1.10-4.34	0.026	3.69	2.13-6.38	<0.001	4.00	1.69-9.46	0.002
Smoking history (+)	2.87	1.85-4.45	<0.001	0.94	0.46-1.93	0.863	2.71	1.62-4.54	<0.001	0.65	0.28-1.51	0.315
CTR	1.02	1.02-1.03	<0.001	1.01	1.00-1.02	0.047	1.02	1.01-1.03	<0.001	1.01	0.99-1.02	0.343
PET maxSUV	1.08	1.05-1.12	<0.001	0.97	0.92-1.02	0.246	1.07	1.02-1.12	0.005	0.95	0.88-1.01	0.111
CEA elevation	2.45	1.64-3.66	<0.001	1.74	1.14-2.66	0.010	2.75	1.73-4.38	<0.001	1.96	1.20-3.21	0.007
Histology (non-AD)	3.84	2.61-5.66	<0.001	1.95	1.22-3.11	0.005	3.51	2.22-5.57	<0.001	1.85	1.05-3.29	0.035
Pleural invasion (+)	3.55	2.30-5.48	<0.001	2.27	1.37-3.74	0.001	2.74	1.58-4.74	<0.001	2.04	1.07-3.87	0.030
Wedge resection	2.10	1.44-3.05	<0.001	1.46	0.99-2.16	0.059	1.83	1.17-2.84	0.008	1.14	0.71-1.81	0.590

AD, adenocarcinoma; CI, confidence interval; CTR, consolidation tumor ratio; HR, hazard ratio; PET, positron emission tomography; SUV, standardized uptake value.

**Figure 3:**
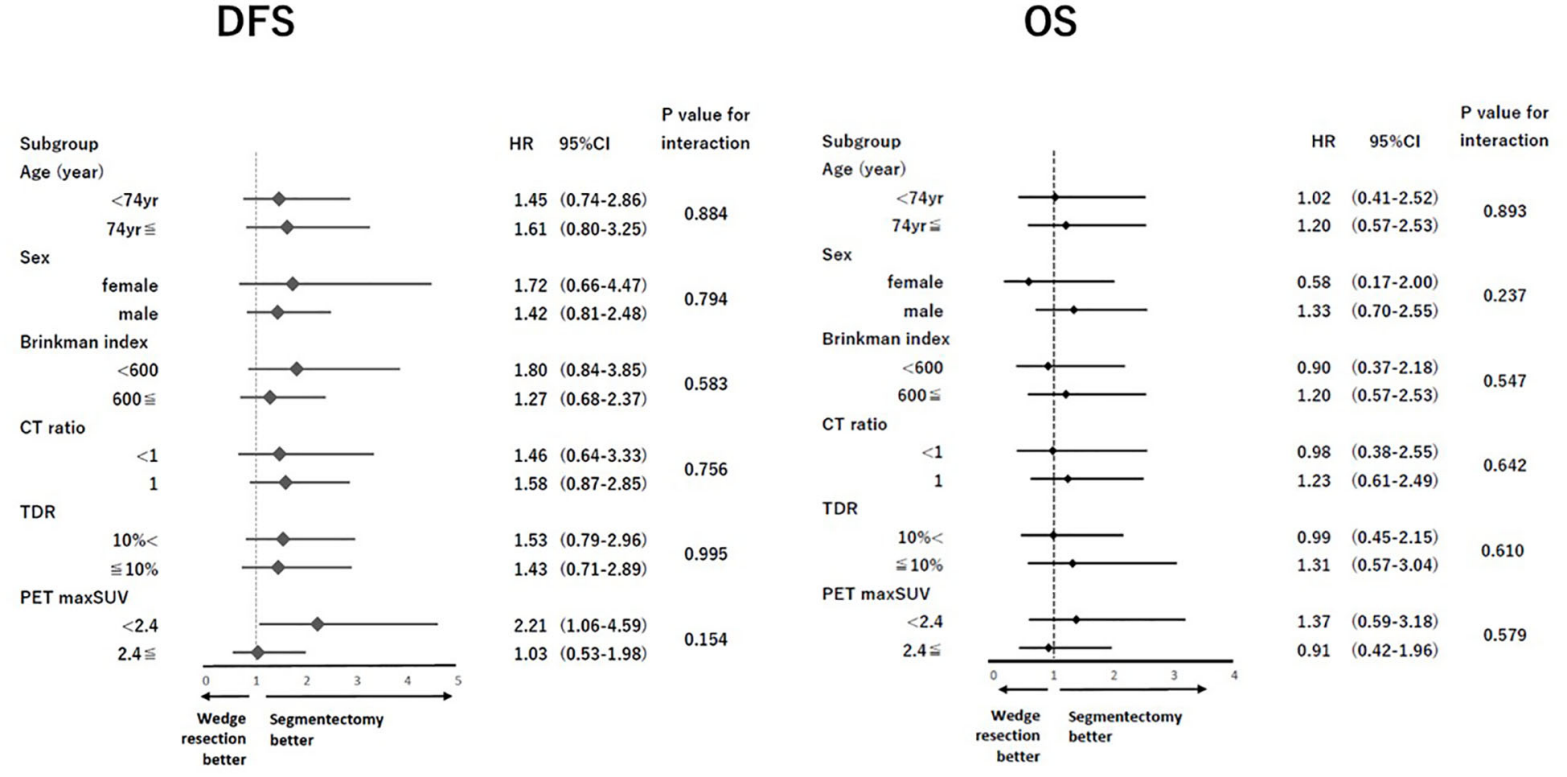
Subgroup analysis for DFS and OS. DFS, disease-free survival; OS, overall survival.

After PSM, the clinical characteristics of the 182 patients in the segmentectomy and wedge resection groups were well matched (**Table 3**). The pathological characteristics of both groups were generally similar. However, the frequencies of pleural invasion and intrathoracic recurrence appeared to be higher in the wedge resection group compared to the segmentectomy group, through these differences were not statistically significant (**Table 3**). There was no significant difference in the DFS between patients who underwent wedge resection and segmentectomy (5-year DFS: 79.9% vs. 87.1%, p = 0.103; **Figure 2C**). Moreover, there was no significant difference in the OS of patients who underwent wedge resection and segmentectomy (5-year OS: 88.7% vs. 88.9%, p = 0.719; **Figure 2D**). As depicted in **Figure 3**, no specific subgroups were identified in which wedge resection or segmentectomy significantly improved DFS or OS.

**Table 3:** Comparison of clinicopathological characteristics between segmentectomy and wedge resection groups after propensity score matching.

Total n=364	Segmentectomy (n=182)	Wedge resection (n=182)	p value	SMD
Age (74y≤), No. (%)	62 (34.1)	63 (34.6)	1.000	0.012
Male, No. (%)	95 (52.2)	95 (4101)	1.000	<0.001
Brinkman index, No. (%) 0 1-600 601-1200 1201-	83 (45.6) 32 (17.6) 41 (22.5) 26 (14.3)	80 (44.0) 32 (17.6) 50 (27.5) 20 (11.0)	0.636	0.138
Right side, No. (%)	91(50.0)	89 (48.9)	0.917	0.022
CT tumor size 1.6cm≤, No. (%)	52 (28.6)	53 (29.1)	1.000	0.012
CTR, No. (%) 26-50 51-99 100	(28.6) 49 (26.9) 81 (44.5)	49 (26.9) 55 (30.2) 78 (42.9)	0.799	0.074
Tumor disappearance rate (10%≥), No. (%)	53 (29.1)	53 (29.1)	1.000	<0.001
PET maxSUV (2.4≤), No. (%)	43 (23.6)	42 (23.1)	1.000	0.013
CEA elevation, No. (%)	26 (14.3)	30 (16.5)	0.663	0.061
Clinical stage IA2≤, No. (%)	86 (47.3)	92 (50.5)	0.600	0.066
Adenocarcinoma, No. (%)	155 (85.2)	146 (80.2)	0.268	
Ly (+), No. (%)	20 (11.0)	16 (8.8)	0.599	
V (+), No. (%)	22 (12.1)	25 (13.7)	0.755	
Pleural invasion, No. (%)	10 (5.5)	20 (11.0)	0.085	
Pulmonary metastasis, No. (%)	2 (1.1)	4 (2.2)	0.685	
Pathological T1c≤, No. (%)	24 (13.2)	35 (19.2)	0.155	
Pathological stage IA3≤, No. (%)	26 (14.3)	35 (19.2)	0.261	
Intrathoracic only, No. (%)	11 (6.0)	22 (12.1)	0.067	
Lung	8 (4.4)	10 (5.5)	0.810	
Lymph node	3 (1.6)	6 (3.3)	0.502	
Surgical margin	0	3 (1.6)	0.248	
Pleura	2 (1.1)	0	0.499	
Extrathoracic (and/or intrathoracic), No. (%)	1 (0.5)	5 (2.7)	0.215	

CEA, carcinoembryonic antigen; CT, computed tomography; CTR, consolidation tumor ratio; Ly, lymphatic vessel invasion; PET, positron emission tomography; SMD, standardized mean difference; SUV, standardized uptake value; V, blood vessel invasion.

As shown in **Supplementary Figure B**, the OS curves adjusted by IPTW showed comparability between the segmentectomy and wedge resection groups after IPTW adjustment (p = 0.165). The adjusted HR for OS with wedge resection on OS was 1.38 (95% CI, 0.88–2.18; p = 0.200). However, the DFS curves adjusted by IPTW were significantly worse in the wedge resection group than in the segmentectomy group (p = 0.013). The adjusted HR of wedge resection was 1.63 (95% CI, 1.10–2.41; p = 0.020).

**Figure 4:**
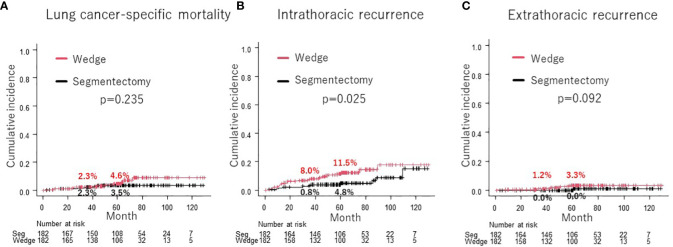
Comparison of cumulative incidence of lung cancer-specific mortality between wedge resection group and segmentectomy group **(A)**. Comparison of cumulative incidence of intrathoracic recurrence **(B)** and extrathoracic recurrence **(C)** between wedge resection group and segmentectomy group.

Cumulative incidence of lung cancer-specific mortality was not significantly different between wedge resection and segmentectomy groups after PSM (4.6% vs. 3.5% at 5-year, p = 0.235; **Figure 4A**). The cumulative incidence of intrathoracic recurrence (11.5% vs. 4.8% at 5-year, p = 0.025; **Figure 4B**) was significantly higher in the wedge resection group than in the segmentectomy group. However, there was no significant difference in the cumulative incidence of extrathoracic recurrence (3.3% vs. 0% at 5-year, p = 0.092; **Figure 4C**).

## 4 Discussion

This large-scale multicenter PSM analysis demonstrated the comparable DFS, OS, and lung cancer-specific mortality of wedge resection and segmentectomy for patients between wedge resection and segmentectomy for cN0 NSCLC ≤ 2 cm in size with CTR > 0.25. Moreover, wedge resection was not a prognostic factor in the multivariable analysis, and there was no specific subgroup with improved DFS or OS by wedge resection or segmentectomy. Although the cumulative incidence of intrathoracic recurrence was higher in the wedge resection group than in the segmentectomy group, that of extrathoracic recurrence was statistically comparable.

A meta-analysis comparing wedge resection and segmentectomy for stage I (7th TNM (25)) reported that segmentectomy resulted in better OS and lung cancer-specific survival (LCSS) (26, 27). In stage IA (7th TNM) NSCLC, patients who underwent segmentectomy had better OS than those who underwent wedge resection (16, 22), although there are reports of comparable OS (9, 11). Moreover, in cN0 NSCLC of ≤ 2 cm in size, a previous study reported that segmentectomy was associated with better OS than wedge resection (22), while others reported that both procedures are comparable in terms of OS (4, 21). The difference in OS efficacy between wedge resection and segmentectomy for early-stage NSCLC remains unclear.

This is the first study to compare the prognosis of patients with cN0 NSCLC ≤ 2 cm in size with CTR > 0.25 who underwent wedge resection versus segmentectomy using PSM analysis, showing no difference in OS between the two techniques. Moreover, no specific subgroup that exhibited enhanced OS following either wedge resection or segmentectomy was found (**Figure 3**). Only a limited number of studies have conducted comparison of the efficacy of wedge resection and segmentectomy for cN0 NSCLC using PSM, and even fewer for tumors of size ≤ 2 cm (**Table 4**). Tsutani et al. compared the prognosis of patients with c-stage I (7^th^ TNM) NSCLC who underwent wedge resection and segmentectomy using PSM analysis and demonstrated comparable OS (segmentectomy, HR = 1.21, p = 0.62) between the two procedures (10). Smith CB et al. compared wedge resection with segmentectomy by using PSM analysis for stage IA(7th TNM)NSCLC and found favorable OS of segmentectomy (HR: 0.8, 95% CI: 0.69–0.93) and LCSS (HR: 0.72, 95% CI: 0.59–0.88) (22). Furthermore, they reported segmentectomy was associated with improved OS (HR: 0.81, 95% CI: 0.67–0.99) and LCSS (HR: 0.75, 95% CI: 0.58–0.98) for NSCLC ≤ 2 cm in size (**Table 4**) (22). Zhou et al. compared the prognosis of 100 paired patients with NSCLC who underwent simple segmentectomy and wedge resection for tumor size 2–3 cm with a solid component less than 2 cm in size using PSM analysis. They reported comparable OS between the two surgical procedures (11). Cao et al. reported comparable OS (HR 1.171, p = 0.541) and LCSS (HR 0.745, p = 0.447) between 126 paired matched patients after wedge resection and segmentectomy for tumors 1 cm or smaller; however, for 429 paired NSCLC patients with tumor of 1.1 cm to 2.0 cm in size, wedge resection was inferior to segmentectomy in terms of OS (HR 1.399, p = 0.005) and LCSS (HR 1.704, p = 0.002) (**Table 4**) (19).

**Table 4:** Previous studies comparing the prognosis of patients after segmentectomy and wedge resection for cN0 non-small cell lung cancer with tumor size ≤ 2 cm using propensity score matching analysis.

Authors (Ref No.) (Year)	Matching variable	CTR	Tumor size	Number of paired patients	Outcomes	Seg	Wedge	p values
Cao J (19) (2018)	Age, sex, race, marital status, metropolitan area, region, year, histology, site, size, and grade	All	≦1.0cm 1.1-2.0cm	126 426	OS	1 (Ref) 1 (Ref)	1.171 1.399	0.541 0.005
			≦1.0cm 1.1-2.0cm	126 426	LCSS	1 (Ref) 1 (Ref)	0.745 1.704	0.447 0.002
Smith CB (22) (2011)	Age, sex, race, marital status, location, size, and histology	All	≦2.0cm	Not specified	OS	0.81	1 (Ref)	Significant
Present study (2023)	Age, sex, Brinkman index, side, size, CTR, TDR, PET maxSUV, CEA, and c-stage	0.25<	≦2.0cm	182	OS	5-year OS 88.9%	5-year OS 88.7%	0.719
					DFS	5-year DFS 87.1%	5-year DFS 79.9%	0.103
					LCSM	5-year LCSM 4.6%	5-year LCSM 3.5%	0.235

CEA, carcinoembryonic antigen; CT, computed tomography; CTR, consolidation tumor ratio; DFS, disease-free survival; LCSM, lung cancer-specific mortality; LCSS, lung cancer-specific survival; maxSUV, maximum standardized uptake values; OS, overall survival; PET, positron emission tomography; Ref, reference; Seg, segmentectomy; TDR, tumor disappearance rate.

There is no consensus regarding the difference in DFS between wedge resection and segmentectomy. For stage I (7^th^ TNM) NSCLC, previous studies have reported comparable DFS between wedge resection and segmentectomy (10, 26); however, other studies have reported superior DFS in the segmentectomy group compared to the wedge resection group (27). Comparable DFS were reported between wedge resection group and segmentectomy group for stage IA (7^th^ TNM) NSCLC (9), and for NSCLC with 2 cm or smaller in size (4). In the present study with PSM analysis, comparable OS, DFS, and lung cancer-specific mortality were observed between wedge resection and segmentectomy group among cN0 NSCLC patients with ≤ 2 cm in size with CTR > 0.25. JCOG0802 and JCOG1211 suggested that segmentectomy is the standard procedure for cN0 NSCLC patients with ≤ 2 cm in size with a CTR > 0.25; however, future prospective studies comparing wedge resection and segmentectomy for these tumors are necessary.

Wedge resection is associated with less intraoperative blood loss (9, 10) and a shorter operative time (9, 11) than segmentectomy. Moreover, wedge resection has been reported to have a lower frequency of postoperative complications than segmentectomy (9–12). While segmentectomy requires a higher surgeon’s expertise to avoid intraoperative and postoperative complications, wedge resection, a non-anatomical resection, is relatively easy and safe compared to other types of pulmonary resection (9). Furthermore, recovery of pulmonary functions such as forced vital capacity, forced expiratory volume in 1 second, predicted diffusing capacity of the lung of carbon monoxide percentage, and peak expiratory flow has been reported to be better with wedge resection than with segmentectomy (4, 11). Although neither wedge resection nor segmentectomy has been reported to have a 30-day postoperative mortality rate (9, 13), wedge resection for patients with NSCLC is considered a less invasive procedure than segmentectomy in terms of blood loss, operating time, and postoperative complications.

In this study, the subgroup analysis did not identify any specific subgroup with a statistically significant favorable prognosis after wedge resection or segmentectomy. However, nearly all subgroups tended to exhibit a favorable DFS after segmentectomy compared to wedge resection (**Figure 3**). Moreover, the cumulative incidence of intrathoracic recurrence was higher after wedge resection than after segmentectomy (**Figure 4B**). Previous studies have reported a higher local recurrence rate for wedge resection than for segmentectomy in patients with stage I NSCLC (7th TNM) (17), stage IA NSCLC (7th TNM) (1), and NSCLC 2 cm in size (13). In contrast, another study reported no difference in the frequency of recurrence or recurrence patterns between Stage IA NSCLC (9) and NSCLC ≦ 2 cm in size (4). In the present study, a trend towards improved DFS after segmentectomy, compared with wedge resection, was observed in the PSM analysis (**Figure 2C**), and significantly better DFS was evident in the segmentectomy group in the IPTW analysis (**Supplementary Figure A**). These findings could be attributed to the reduced intrathoracic recurrence post segmentectomy.

There are four possible reasons why intrathoracic recurrence was more common in the wedge resection group. First, although not examined in this study, tumor margins may have been smaller in the wedge resection group than in the segmentectomy group. The incidence of local and intrathoracic recurrence has been reported to be associated with macroscopic parenchymal resection margins (9), and several studies have reported shorter tumor margins with wedge resection than with segmentectomy (9, 10, 12). However, Zhou et al. reported no difference in tumor margins between segmentectomy and wedge resection (19.5 mm vs 22.4 mm, p = 0.7) (11). The appropriate tumor margin to prevent local recurrence *via* sublobar resection for cN0 NSCLC ≦ 2 cm in size with CTR > 0.25 needs further elucidation. Secondly, wedge resection is more difficult than segmentectomy for obtaining intrathoracic lymph nodes, especially those located in the hilar region (9, 11, 13), which may have resulted in increased intrathoracic recurrence after wedge resection. Third, patients who undergo wedge resection may be understaged more than those who undergo segmentectomy because of the difficulty in obtaining intrathoracic lymph nodes during surgery (28). Therefore, these patients may lose the opportunity to receive postoperative adjuvant therapy, which may increase their risk of recurrence. Fourth, there was a trend toward more positive pathological pleural invasion in the wedge resection group than in the segmentectomy group (5.5% vs. 11.0%, p = 0.086). Tumors located closer to the pleura were more common in the wedge resection group, which may have been one reason for the higher cumulative intrapleural recurrence rate in the wedge resection group. Large prospective randomized clinical trials are needed to compare the recurrence rates between wedge resection and segmentectomy.

It is unclear why there was no difference in the OS between the two groups, even though the cumulative intrathoracic recurrence rate was higher for wedge resection in this study. It has been reported that local recurrence after wedge resection is often caused by the remaining lung parenchyma in the former resection area, and local treatment for local recurrence, such as reoperation or radiation therapy, may result in a favorable prognosis even after recurrence (13). In this study, among patients experiencing intrathoracic recurrence, the occurrence of lone intrathoracic lymph node recurrence or lone lung metastasis (excluding cases of multiple lung metastases) was higher in the wedge resection group (54.5%, n = 12) compared to the segmentectomy group (36.4%, n = 4, respectively). Because post-relapse survival after intrathoracic recurrence, including pulmonary metastases and intrathoracic lymph node recurrence, is reported to be favorable (29), there may have been no difference in OS between the two groups, although more intrathoracic recurrences were observed in the wedge resection group.

This study has several limitations. First, this was a retrospective study, and selection bias may have occurred. Although this study is based on a comprehensive multicenter database, it remains uncertain whether the sample size was sufficiently large to definitively conclude that the prognosis of cN0 NSCLC patients with tumor diameters ≦ 2 cm and CTRs > 0.25 is equivalent between wedge resection and segmentectomy. Additional extensive prospective clinical trials are required to effectively compare the prognoses of patients who undergoing wedge resection and segmentectomy. Second, the absence of information on surgical outcomes such as blood loss, operation time, and surgical complications limits the ability to analyze the substantial invasiveness associated with wedge resection and segmentectomy in this study. Moreover, the patient comorbidities and preoperative respiratory function was lacking for preoperative variables in the PSM analysis. Third, this study did not account for factors such as location (peripheral or central), distance from the margin, or the existence of tumor spread through the air space, which have been associated with local recurrence. Fourth, a description of treatment after recurrence was lacking in this study, and the effect of local therapy on patients with intrathoracic recurrence remains unclear.

## 5 Conclusion

In conclusion, this large-scale multicenter PSM analysis demonstrated the comparable DFS, OS, and lung cancer-specific mortality of patients who received wedge resection and segmentectomy for cN0 NSCLC ≤ 2 cm in size with CTR > 0.25. Large-scale prospective clinical trials are warranted to compare the prognoses of wedge resection and segmentectomy for these tumors.

## Data availability statement

The raw data supporting the conclusions of this article will be made available by the authors, without undue reservation.

## Ethics statement

The studies involving humans were approved by Kanagawa Cancer Center, Tokyo Medical University Hospital, and Hiroshima University Hospital. The studies were conducted in accordance with the local legislation and institutional requirements. Written informed consent for participation was not required from the participants or the participants’ legal guardians/next of kin because The institutional review board of the participating institutions has approved this retrospective review of the multicenter database and waived the requirement for informed consent for each patient (Kanagawa Cancer Center, approval 24EKI54 (Approved on June 14, 2021); Tokyo Medical University Hospital, approval SH2969; Hiroshima University Hospital, approval E-1216).

## Author contributions

TI: Conceptualization, Data curation, Formal Analysis, Investigation, Methodology, Software, Validation, Visualization, Writing – original draft, Writing – review & editing. TN: Data curation, Supervision, Writing – review & editing. HA: Data curation, Supervision, Writing – review & editing. HN: Methodology, Software, Supervision, Validation, Writing – review & editing. KM: Data curation, Writing – review & editing. SS: Data curation, Writing – review & editing. NK: Data curation, Writing – review & editing. NS: Data curation, Writing – review & editing. KW: Data curation, Writing – review & editing. YK: Data curation, Writing – review & editing. YM: Data curation, Writing – review & editing. MO: Data curation, Supervision, Writing – review & editing. NI: Data curation, Supervision, Writing – review & editing. HI: Conceptualization, Data curation, Supervision, Writing – review & editing.

## Supplementary material

Supplementary Figure Comparison of DFS (Supplementary Figure A) and OS (Supplementary Figure B) between the wedge resection group and the segmentectomy group after inverse probability of treatment weighting (IPTW) analysis, based on propensity scoring. DFS, disease-free survival; IPTW, inverse probability of treatment weighting; OS, overall survival.
