# Supplementary figures and images for: Wedge resection vs. segmentectomy for lung cancer measuring ≤ 2 cm with consolidation tumor ratio > 0.25
Source: Front Oncol. 2023 Aug 28;13:1253414. doi: 10.3389/fonc.2023.1253414 (PMC10493869; doi:10.3389/fonc.2023.1253414)

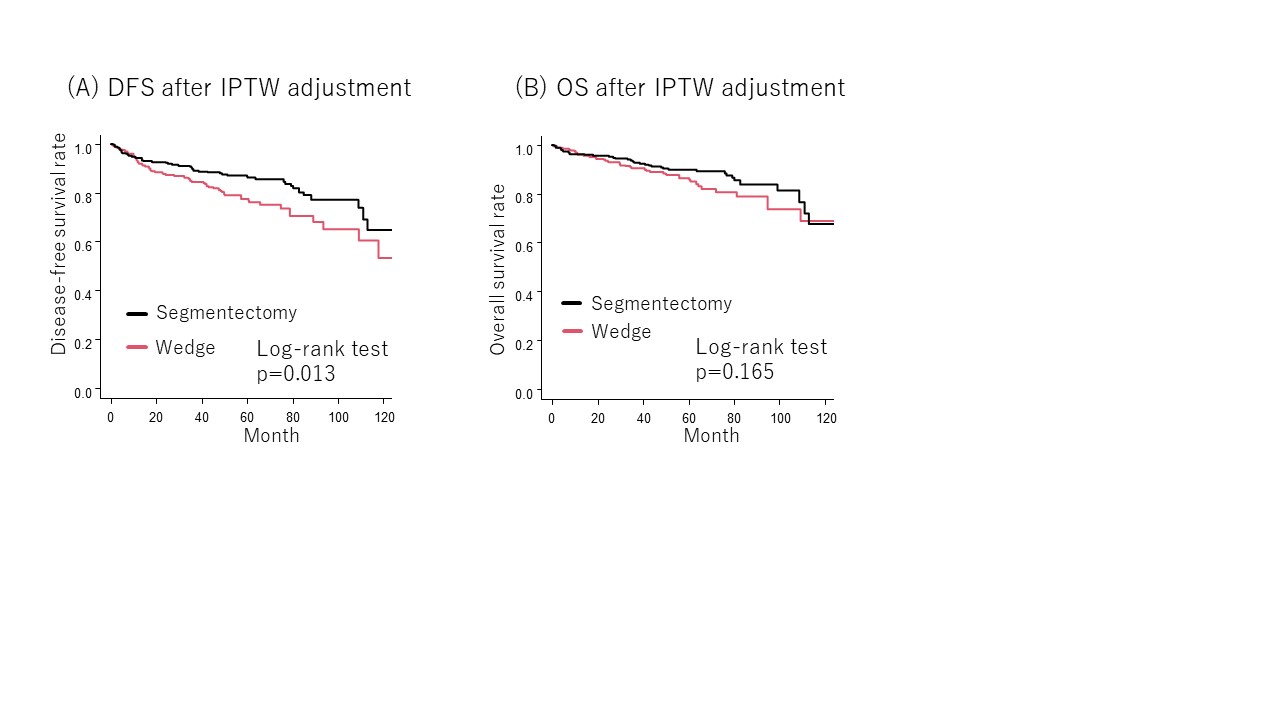

Supplement: Supplementary Figure — Comparison of DFS (Supplementary Figure A) and OS (Supplementary Figure B) between the wedge resection group and the segmentectomy group after inverse probability of treatment weighting (IPTW) analysis, based on propensity scoring. DFS, disease-free survival; IPTW, inverse probability of treatment weighting; OS, overall survival. [file Image_1.jpeg]
